# Model-driven design allows growth of *Mycoplasma pneumoniae* on serum-free media

**DOI:** 10.1038/s41540-020-00153-7

**Published:** 2020-10-23

**Authors:** Erika Gaspari, Antoni Malachowski, Luis Garcia-Morales, Raul Burgos, Luis Serrano, Vitor A. P. Martins dos Santos, Maria Suarez-Diez

**Affiliations:** 1 Laboratory of Systems and Synthetic Biology, Wageningen University and Research, Wageningen, the Netherlands; 2 INRA, UMR 1332 de Biologie du Fruit et Pathologie, F-33140 Villenave d’Ornon, France; 3 Centre for Genomic Regulation (CRG), The Barcelona Institute for Science and Technology, Doctor Aiguader 88, Barcelona, 08003 Spain; 4 Universitat Pompeu Fabra (UPF), Barcelona, Spain; 5 Institució Catalana de Recerca i Estudis Avançats (ICREA), Pg. Lluis Companys 23, Barcelona, 08010 Spain; 6 LifeGlimmer GmbH, MMarkelstrasse 38, Berlin, Germany; 7 Present Address: Laboratory of Systems and Synthetic Biology, Wageningen University and Research, Wageningen, The Netherlands

**Keywords:** Biochemical networks, Computational biology and bioinformatics, Metabolic engineering, Computer modelling

## Abstract

*Mycoplasma pneumoniae* is a slow-growing, human pathogen that causes atypical pneumonia. Because it lacks a cell wall, many antibiotics are ineffective. Due to its reduced genome and dearth of many biosynthetic pathways, this fastidious bacterium depends on rich, undefined medium for growth, which makes large-scale cultivation challenging and expensive. To understand factors limiting growth, we developed a genome-scale, constraint-based model of *M. pneumoniae* called iEG158_mpn to describe the metabolic potential of this bacterium. We have put special emphasis on cell membrane formation to identify key lipid components to maximize bacterial growth. We have used this knowledge to predict essential components validated with in vitro serum-free media able to sustain growth. Our findings also show that glycolysis and lipid metabolism are much less efficient under hypoxia; these findings suggest that factors other than metabolism and membrane formation alone affect the growth of *M. pneumoniae*. Altogether, our modelling approach allowed us to optimize medium composition, enabled growth in defined media and streamlined operational requirements, thereby providing the basis for stable, reproducible and less expensive production.

## Introduction

Mycoplasma are a small genus of bacteria belonging to the Mollicute class^1^, comprising 124 species, 14 of which are human pathogens^2^, while others infect farm animals, herd animals and pets^3^. Most large-scale animal farms make use of antibiotics to fight and prevent infections, which often lead to development of antibiotics-resistant bacteria^4–6^. Moreover, chronic infections caused by several Mycoplasmas are not immediately detectable. Therefore, the use of effective vaccines against various Mycoplasmas in farm animals might be a solution to prevent wide spreading of these chronic infections^7^. It has been suggested that, despite being primarily considered a human pathogen, *Mycoplasma pneumoniae* could potentially be used as a universal chassis to be deployed as single-vaccine or multi-vaccine in a range of animal hosts (www.mycosynvac.eu).

*M. pneumoniae* causes atypical or walking pneumonia^8^. With its highly reduced genome (816 kb), it is among the smallest self-replicating living organisms and has an exclusively parasitic lifestyle. This feature greatly determined its evolution towards minimal functions (e.g., absence of vitamins and lipids synthesis) and inability to detoxify metabolic waste (e.g., hydrogen peroxide), tasks for which this bacterium fully relies on the host^9^. *M. pneumoniae* primarily ferments glucose^10^ and uses most of its energy (71 to 88%) for non-growth associated tasks^11^. One of the key features of this pathogen during infections is its ability to evade the immune system: the unique characteristics of its membrane (in particular the high cholesterol contents) allows the mimicking of the host membrane^12^. Moreover, the lack of a cell wall makes it resistant to antibiotics which target this structure^13^.

The development of vaccines based on *M. pneumoniae* requires the large-scale production of this bacterium in a serum-free defined medium. This is due to the high economic cost of rich media, the variable composition of animal-derived compounds^14^ and the possible presence of toxins, viruses or antigens that could reduce the sensitivity of immunological assays used on farms to screen for infection^15^. However, the production of *M. pneumoniae* in serum-free media is a major challenge. For decades, the ability to culture *M. pneumoniae* was reported to be possible only in serum-rich media^16^ due to a requirement for sterol components^17^. Only in 2009 Yus et al. reported a defined medium based on the metabolic map reconstruction of *M. pneumoniae* that allowed some growth of the bacterium but required daily changes of the medium^18^. This medium is not valid for large-scale growth needed during vaccine production. For these reasons, we focused our study on the model-driven design of a serum-free medium that could improve *M. pneumoniae* growth rate.

We approached the study through metabolic modeling, a method widely used for genome-scale biochemical networks analysis^19–26^. We previously described the development of a genome-scale, constraint-based model (GEM) of *M. pneumoniae* metabolism, iJW145^11^, and its use to carry out predictions related to growth rate and medium design, relying upon a biomass composition that had been formulated from literature^3^, sequencing, proteomics and mass spectrometry data. However, no novelty in the defined medium composition was reported^11^. Several studies have shown that lipids are among the most important component limiting growth, mainly cholesterol, regulating membrane fluidity (i.e., viscosity of the lipid bilayer), and fatty acids^1,27–30^. Determining the lipids composing the membrane of *M. pneumoniae* is challenging, as it is strongly dependent on the composition of the growth medium, growth phase and culture conditions^31^. Some key features remain invariant, such as the high cholesterol proportion, directly incorporated from the medium^32^. The model iJW145 did not account in detail for lipids in its biomass composition^11^. Therefore, we integrated native lipid pathways of *M. pneumoniae*, and reactions involved in the membrane formation to develop an updated GEM of M. pneumoniae metabolism, called iEG158_mpn.

We aimed to describe the use of the energy available, considering not only cytosolic processes but also transmembrane transport. We used the model iEG158_mpn to predict the essentiality and synergism of components in serum-free media for *M. pneumoniae* and to study how certain culture conditions may affect the growth of this bacterium, focusing on its intracellular metabolism. These predictions were corroborated with defined-growth media that support robust growth (up to 60% of the biomass obtained with rich medium) and shows the synergism of components. Our study, in combination with the existing literature, suggests membrane formation and adaptability are key aspects to be investigated in relation to *M. pneumoniae* growth, which appears to be limited by several factors.

## Results

### Features of iEG158_mpn

The model iEG158_mpn of *M. pneumoniae* metabolism has two compartments, one extra-cellular and one cytosolic, and consists of 490 reactions, 442 metabolites and 158 genes. Of the total reactions, 314 are gene-associated, 329 are cytosolic conversion reactions, 65 represent transport between the extracellular compartment and the cytosol and vice versa, and 96 are of metabolite exchange, meaning they allow uptake/production of compounds. Only 61 of the cytosolic reactions have a zero-flux range, meaning they are not active under the simulated conditions. Excluding the zero-flux reactions, 293 reactions have same minimum and maximum fluxes according to FVA, meaning in total 354 reactions with fixed flux. The high proportion of active reactions and the lack of flexibility in the usage of alternative reactions reflect the evolution of the organism in minimal genome. In the biomass equation, formulated according to Wodke et al.^11^, cofactors and vitamins, known to be essential for growth but with no available measurements, were symbolically included as traces in the biomass equation. Similarly, DNA repair and RNA turnover were considered in the biomass composition by slightly increasing their proportions. Protein turnover is modeled by the addition of degradation reactions^33^, which is reflected in iEG158_mpn as the decomposition of a general protein compound into amino acids with the associated ATP cost.

**Fig. 1:**
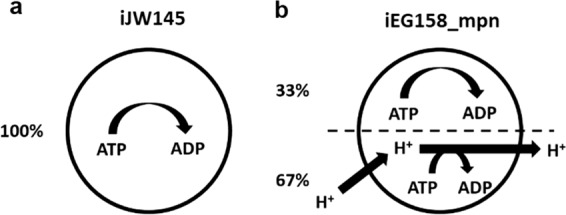
Representation of maintenance energy exploitation in two GEMs of *M. pneumoniae*. **a** Maintenance energy in model iJW145 and (**b**) Maintenance energy in model iEG158_mpn. In iEG158_mpn simulations, the ATPase is observed to run in reverse to allow efflux of protons for cytosol de-acidification. Maintenance reaction in iEG158_mpn was therefore updated to explicitly include a proton leakage component (67%).

The very small size of *M. pneumoniae* contributes to the increase in maintenance costs, as the high surface-to-volume ratio induces higher proton leakage through the membrane. The ATP maintenance reaction was determined in iJW145 by multiplying the number of ATPase complexes on the cell surface by the number of cells in a gram of biomass, so considering only the cytosolic side of the energy requirement. However, the reaction fluxes representation allowed the visualization of a flux of protons out of the cell, required for cytosol de-acidification. Moreover, it is stated in Wodke et al.^11^ that the proportion of ATP used for cellular tasks not directly related to growth (which corresponds to total non-growth associated maintenance energy in iJW145) is about 76% at 24 h after inoculation. We updated the non-growth-associated maintenance reaction (model reaction ID: “ATPM”) to specifically include proton leakage component (66% of the total ATP consumption not associated to growth) (model reaction ID: “protonLeak”), as represented in Fig. 1. Furthermore, this introduced a link between the non-growth associated maintenance equation and the proton production/consumption reactions.

The maintenance reaction in iEG158_mpn was further updated according to the results of the sequence alignment for monocarboxylic acids transporters discovery: no lactate or acetate proton symporter were identified to be present in *M. pneumoniae* genome. The respective transport reactions (IDs “*LACLt”* and “*ACtr”*) were therefore updated in iEG158_mpn as irreversible, removing the proton symport and the maintenance costs adjusted to the maximum value allowing growth, computed as 10.46 mmol.g_DW_^−1^.h^−1^ of ATP (g_DW_ = grams dry weight) used for non-growth-associated tasks at the quasi-steady state (24 h time point).

Assuming the total maintenance cost, Mt, equals to 10.46 mmol.g_DW_^−1^.h^−1^ of ATP, the energy fraction spent for proton leakage, EP, equals to 0.67 and the fraction spent on other maintenance requirements, EL, equals 0.33. Therefore, the lower bounds PLB and LLB of the correspondent reactions are computed as:

1 $$P_{{\mathrm{LB}}} = M_t.E_P.4$$

2 $$L_{{\mathrm{LB}}} = M_t.E_L$$

*P*_LB_ is multiplied by 4 as the ATPase reaction removes four protons at a time.

We simulated the growth rate of *M. pneumoniae* with iEG158_mpn supplementing in silico the medium predicted with model iJW145: the resulting growth rate is 0.053 divisions per hour at quasi-steady state of MPN (24 h), correspondent to a doubling time of 13.4 h, in line with the experimental evidences.

### Reconstruction of membrane lipids in iEG158_mpn

Because membrane adaptability is one of the key features of *M. pneumoniae*, we assumed that accounting for membrane formation in the model would give important insights into its growth requirements. Focusing on membrane lipid components, we included in iEG158_mpn the pathways for lipids assembly^34^ and reconstructed the membrane lipid composition through an extensive literature search. Articles listed in this section were used to determine lipids percentages in the membrane and their acyl chains composition. Only lipids whose percentage in the membrane has been reported to be higher than 1% of the total lipid biomass were considered. Presence and proportions of the lipids are strongly dependent on the compound’s availability in the medium, therefore quantities must be interpreted as ranges.

One of the key features of the membrane of *M. pneumoniae* is the high proportion of cholesterol, from 35 to 50% of the total lipid fraction^35^. In vitro, cholesterol is directly incorporated from the medium without being processed by the bacterium. For this reason, in iEG158_mpn, cholesterol has been included into the biomass composition without being metabolically processed. Available literature indicates it constitutes about one third of the final membrane and about half of the lipid components of the membrane.

Most of the studies on *M. pneumoniae* utilize Hayflick media, composed of 16% of horse serum, rich in sphingolipids and phosphatidylcholine^36^. Sphingolipids-phosphatidylcholine ratio in *M. pneumoniae* is 2.4^37^, constituting on average 12 and 5% of the membrane lipids, respectively^38^. However, sphingolipids have a good affinity for cholesterol, while phosphatidylcholine repels it, meaning their proportions in the membrane are strongly correlated to the amount of cholesterol directly incorporated from the medium: a higher amount of cholesterol leads to the incorporation of a higher fraction of sphingolipids and a lower fraction of phosphatidylcholine^39^. The interrelation between these two lipids suggests their essentiality in the membrane is strongly related to their fatty acid chains, being the only lipids carrying the unsaturated fatty acid chain C18:2, whose presence might be determinant in *M. pneumoniae* membrane. Moreover, sphingomyelin incorporated into the membrane has a lower proportion of fatty acid chains with 20 carbons or more in respect to the sphingolipids in the serum^40^, suggesting *M. pneumoniae* preferentially incorporates in the membrane sphingomyelin with less than 20 carbons-fatty acid chains. *M. pneumoniae* is a fatty acid and sterol auxotroph, but it can assemble phosphatidic acid, diacylglycerol, phosphatidylglycerol and glycolipids from provided fatty acids in the medium^34^. Glycolipids constitute a range of 5–15% of the total membrane lipids^41^, while the remaining percentage should be constituted by phosphatidylglycerol; phosphatidic acid and diacylglycerol, being lipid synthesis intermediaries, are not found in relevant proportions in the membrane.

The lipids, with their specific molecular weight, were included in the biomass equation to reflect the predicted membrane composition. We assumed a lipid composition is optimal when it confers optimal fluidity, and so permeability, to the membrane of *M. pneumoniae*, where for optimal fluidity it is meant the combination of thickness and viscosity that allows *M. pneumoniae* to perform its membrane-related activity. While the optimal thickness is considered to be given by a range of lipid proportions, i.e., measured when the bacterium is grown on rich-medium with full metabolites and lipids availability, the optimal viscosity is given by the fatty acids distribution that *M. pneumoniae* aims at incorporating in the membrane when lipids availability is limited. Therefore, the acyl chains of the lipids constitute a critical aspect of our study.

With the aim of linking the membrane lipid profile to the availability of fatty acids in the medium, we reconstructed the acyl chains of the various lipids composing the membrane by considering the sum of fatty acid chain fractions previously determined by Wodke et al.^11^ and Worliczek et al.^40^; a comparison of the total fatty acid composition between iEG158_mpn and the ones available in literature is provided in Supplementary Fig. 1. *M. pneumoniae* tends to selectively incorporate palmitic acid over stearic acid in the membrane^30^, with a preference for saturated fatty acids, suggesting oleic and linoleic acids are incorporated in a lower proportion with respect to the amount found in the media. This indicates that the proportion of different acyl chains for de-novo assembled phosphatidic acids is conserved during assembly of downstream lipids phosphatidylglycerol, diacylglycerol and glycolipids. We assumed phosphatidylcholine could be degraded in silico into glycerol-3-phosphocholine and fatty acids, to represent the re-arrangements of the phospholipid’s acyl chains, which could be triggered by membrane lipases in vivo^42^. Sphingomyelin, on the other hand, is in our model directly incorporated into the membrane without modifications.

**Fig. 2:**
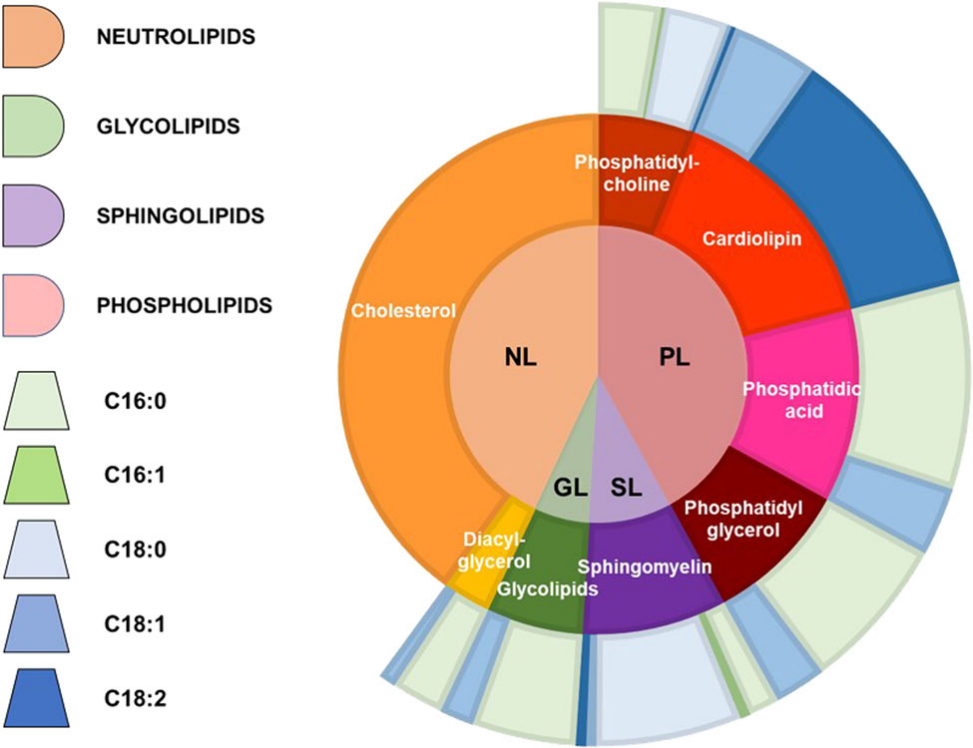
Reconstructed average composition of *M. pneumoniae* membrane lipids and their respective fatty acid chains when the bacterium is grown on a serum-rich medium. The inner circle indicates lipid groups: neutrolipids (NL), glycolipids (GL), sphingolipids (SL) and phospholipids (PL). Lipids are represented in the intermediate circle as belonging to the different groups of the inner one; therefore, PL are constituted by phosphatidylcholine, cardiolipin, phosphatidic acid and phosphatidylglycerol, SL by sphingomyelin, GL by glycolipids and NL by cholesterol and diacyl-glycerol. Each lipid is then represented in the outer circle with their average fatty acid chains composition: the different proportions are made of palmitic acyl chains (C16:0), palmitoleic acyl chains (C16:1), stearic acyl chains (C18:0), oleic acyl chains (C18:1) and linoleic acyl chains (C18:2).

**Fig. 3:**
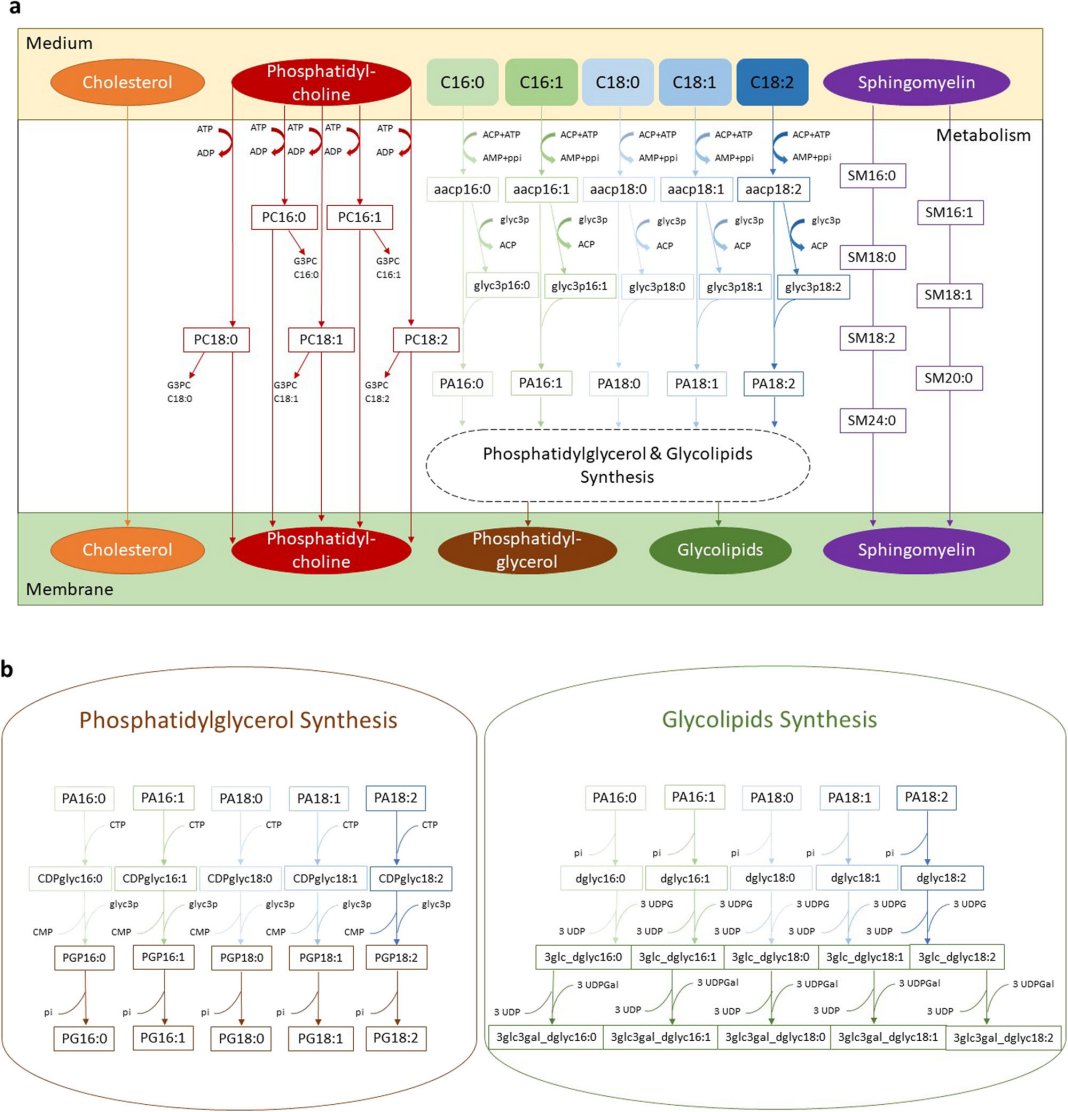
Implementation scheme of the lipid pathways and membrane formation in iEG158_mpn, when *M. pneumoniae* is grown on serum-free medium. The wide variety of lipid species was simplified by considering all carry a representative acyl chain distribution instead of a mix with different acyl chain configurations. This simplification considerably reduces the complexity of the model while at the same time maximizing the amount of quantitative information. **a** Cholesterol is directly incorporated in the membrane, as well as sphingomyelin (SM), for which all the different fatty acid chains proportions have been considered. Phosphatidylcholine is imported using ATP and either goes to build the membrane or it is degraded into its fatty acid chains and glycerol-phosphocholine (G3PC). All the fatty acids introduced in the medium (C16:0, C16:1, C18:0, C18:1, C18:2) will be linked to an acyl-carrier protein (ACP) at the expense of ATP. The ACP then releases the fatty acid chain to glycerol 3-phosphate (glyc3p) and the product, reacting with the ACP carrying fatty acids, leads to the production of phosphatidic acid (PA). **b** PA then undergoes synthesis of phosphatidylglycerol (PG) or glycolipids.

A representative average membrane lipid composition of *M. pneumoniae* grown on serum rich medium is shown in Fig. 2, with its respective and total fatty acid chains. In order to predict in silico the composition of serum-free medium that would maximize the growth of *M. pneumoniae*, we added to the biomass equation the above representative medium composition, excluding those whose supplementation is linked to the addition of serum or would deviate from the established fatty acids proportions (e.g., cardiolipin). We excluded phosphatidic acid from the biomass equation in iEG158_mpn and consider it solely as precursor of phosphatidylglycerol and glycolipids. Figure 3 consists of a visual representation of lipid pathways and membrane construction as they have been implemented in iEG158_mpn. The membrane is then composed of cholesterol, phosphatidylcholine, phosphatidylglycerol, glycolipids and sphingomyelin. We reconstructed their pathways within the metabolism of *M. pneumoniae*.

The possible ranges of lipids and fatty acids proportions assuming growth in serum-free media are summarized in Supplementary Table 1.

### Model simulations predict the addition of two lipids in growth media greatly increases the growth rate of *M. pneumoniae*

We integrated into iEG158_mpn reactions involved in fatty acids, lipids and membrane formation pathways to reconstruct the membrane of *M. pneumoniae* and predict essential fatty acids and lipids that, if added to the medium, could allow growth. The lipids in the biomass reaction of iEG158_mpn have been distributed as reported in Table 1.

**Table 1:** Lipid proportions in terms of percentage of total biomass and percentage of total biomass lipids.

Lipids	% of total biomass	% of total biomass lipids
Glycolipids	2.46	5.82
Cholesterol	18.13	42.92
Phosphatidylcholine	2.58	6.11
Phosphatidylglycerol	12.93	30.62
Sphingomyelin	6.14	14.53

**Fig. 4:**
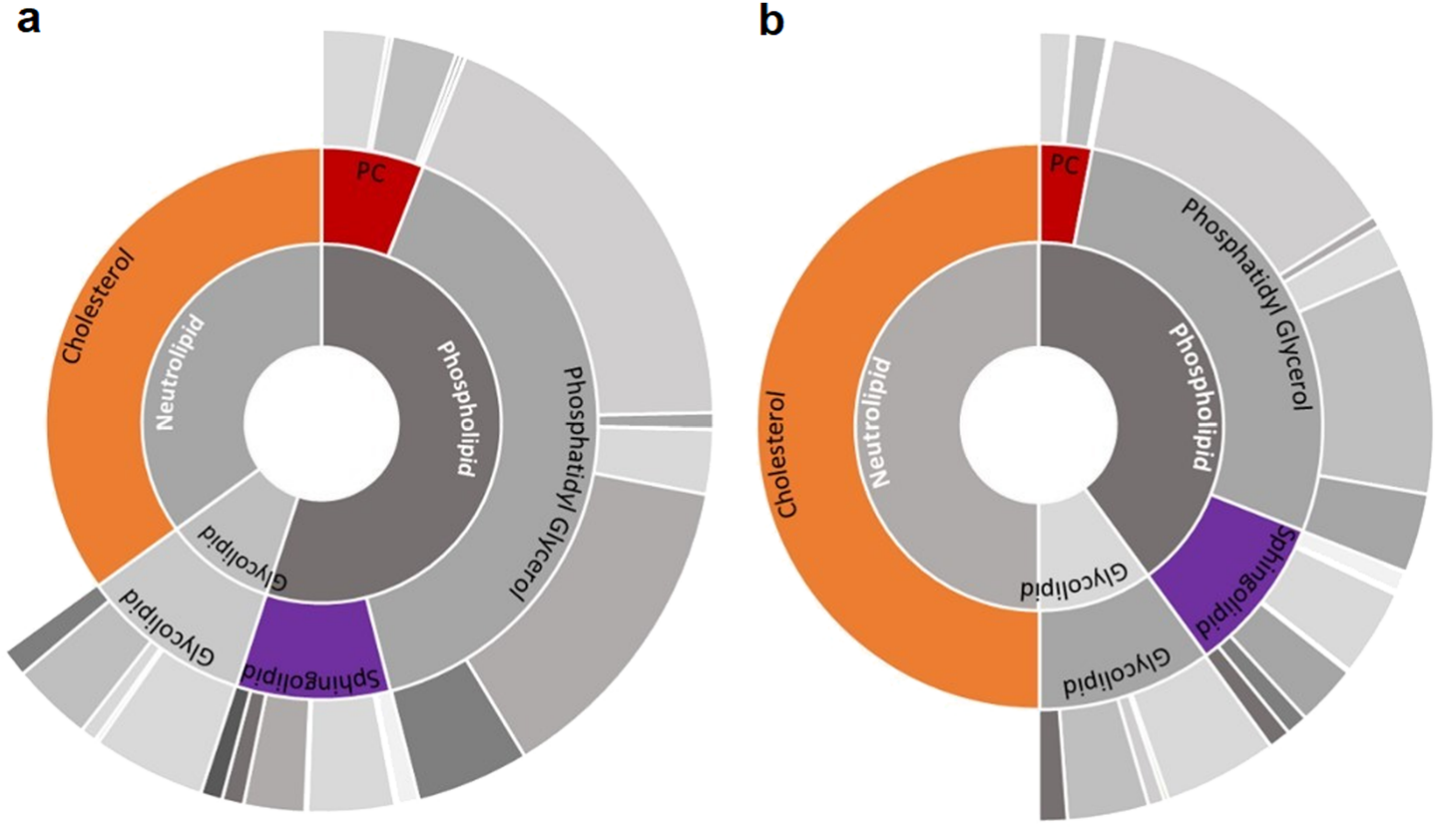
Interrelation between cholesterol, sphingomyelin and phosphatidylcholine proportions in the membrane of *M. pneumoniae* as simulated by iEG158_mpn when grown in silico on the predicted optimal serum-free medium. **a** If cholesterol constitutes 35% of the total membrane lipids, phosphatidylcholine (PC) proportion is up to 10%, while sphingomyelin/sphingolipids is reduced at its minimum to 12%. **b** If cholesterol constitutes 50% of the membrane lipids, phosphatidylcholine (PC) proportion decreases to 6%, while sphingomyelin increases to 15%. Outer cycle represents the average proportions of acyl chains carried by each lipid, as also showed in Fig. 3: the gray colors represent, in order of shading from the lightest to the darkest, C16:0, C16:1, C18:0, C18:1, C18:2.

iEG158_mpn was used to identify media components for increased growth, predicting all the components already highlighted in Yus et al.^18^, with addition of sphingomyelin and phosphatidylcholine. Our analysis pin-points the synergy between the three key lipid components (cholesterol, sphingomyelin and phosphatidylcholine) that were integrated in iEG158_mpn, in terms of percentages of total lipid mass in the membrane, as follows: the most enriched components must be cholesterol, constituting a range of 35–50% of the total lipids; sphingomyelin and phosphatidylcholine proportions are adjusted accordingly, as sphingomyelin has high affinity for cholesterol, while phosphatidylcholine has a low one^39^. Therefore, they constitute, respectively, 9–15% and 6–10% of the total lipids. Figure 4 shows the interrelation between cholesterol, phosphatidylcholine and sphingomyelin (or in general sphingolipids) proportions in the membrane of *M. pneumoniae* as simulated by iEG158_mpn. Sphingomyelin is preferably incorporated carrying an 18-carbon acyl chain and, therefore, its essentiality might be linked to it being the only one carrying the acyl chain C18:2.

The essential fatty acids to be incorporated in a serum-free medium are palmitic (C16:0) and oleic (C18:1) acids, as they are the preferred components of phosphatidic acid in *M. pneumoniae* and its downstream products phosphatidylglycerol and glycolipids. Stearic acid (C18:0) presence is important depending on phosphatidylcholine proportion: once phosphatidylcholine is incorporated, its unsaturated fatty acid chains are replaced with saturated ones, resulting in the membrane di-saturated phosphatidylcholine^43^. Moreover, supplementation of phosphatidylcholine might be important not only as membrane component but also as source of those fatty acids whose presence has been detected in lower percentage with respect to palmitic and oleic acids: linoleic acid (C18:2) and palmitoleic acid (C16:1).

### The developed serum-free semi-defined medium MCMyco supports robust growth of *M. pneumoniae*

Following the model predictions, we have added sphingomyelin and phosphatidylcholine to the so-called MC medium, previously used for growing *M. florum*^44^. Cholesterol and fatty acids were added as previously reported in Yus et al.^18^ to be essential for *M. pneumoniae* growth. The new developed medium has been called MCMyco and its composition consists of Mycoplasma broth base supplemented with 17% yeast extract 15% Solution (Gibco), 0.5% glucose, 500 U/ml of penicillin G and 0.002% phenol red. Delipidated Bovine Serum Albumin was added at a final concentration of 0.5% premixed with the lipid components predicted to be essential by model simulations: fatty acids, cholesterol, sphingomyelin and phosphatidylcholine. We refer to this medium as “semi-defined” due to the presence of the undefined component yeast extract in the MC base. However, yeast extract is reported to contain amino acids, carbohydrates, vitamins and minerals.

**Fig. 5:**
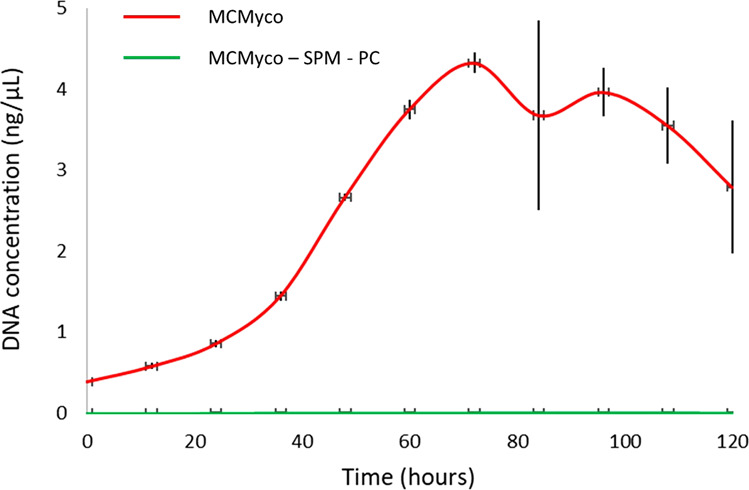
Growth of *M. pneumoniae* strain M129 on serum-free semi-defined medium MCMyco measured by genomic DNA quantification after three culture passages. Growth is detected when *M. pneumoniae* is grown on full MCMyco medium (red) and in the same medium after removing both sphingomyelin (SPM) and phosphatidylcholine (PC) (green). In absence of the two phospholipids, *M. pneumoniae* growth is undetectable. Measurements represent an average of 6 replicates and error bars represent the standard deviations of the 6 replicates.

MCMyco was used to grow *M. pneumoniae* strain M129, resulting in a doubling time of 17.6 h after three culture passages, compared to the doubling time of about 8 h obtained in rich media^18^. The importance of sphingomyelin and phosphatidylcholine is hereby confirmed by the absence of *M. pneumoniae* growth when the two phospholipids are removed from MCMyco (Fig. 5).

### Importance of phosphatidylcholine and sphingomyelin for growth of *M. pneumoniae* into the serum-free defined medium vB13

The defined serum-free medium for *M. pneumoniae* published by Yus et al.^18^ only supports very poor growth compared to Hayflick. Therefore, some of the authors of this work, Burgos R. and Serrano L., together with Garcia-Ramallo E., Shaw D., and Lluch-Senar M. improved this medium developing a new serum-free defined medium called vB13, resulting in about 60% of the biomass obtained in Hayflick (Supplementary Fig. 2).

**Fig. 6:**
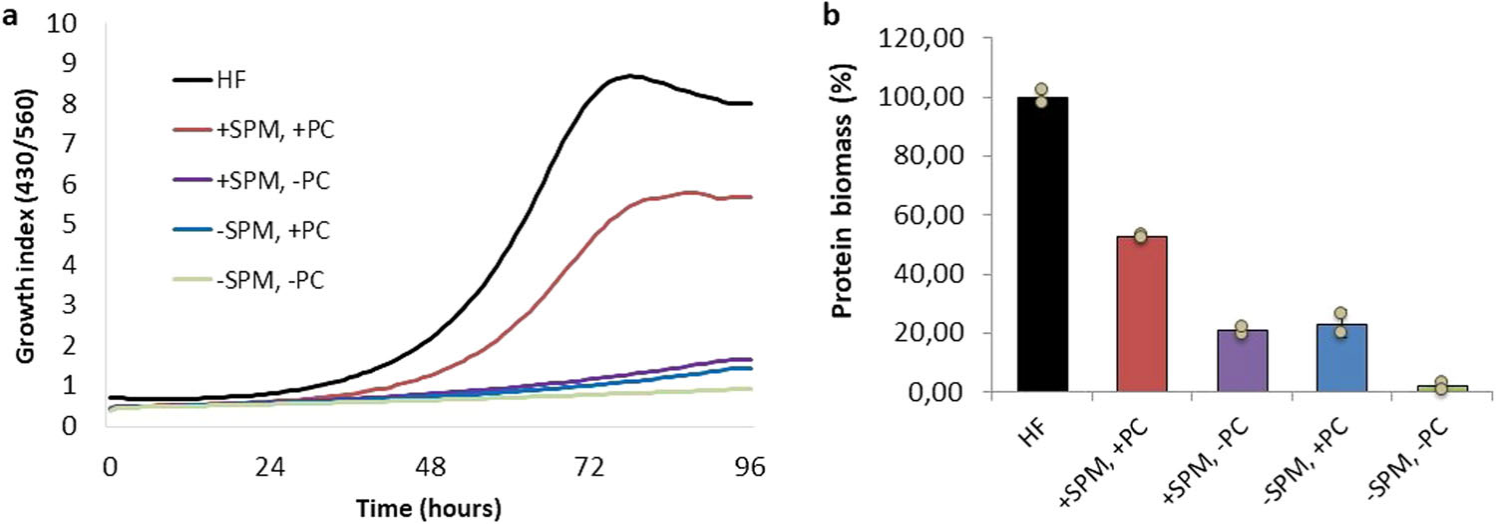
Impact of phosphatidylcholine and sphingomyelin on *M*. *pneumoniae* strain M129 cell growth using the serum-free vB13 medium. **a** Growth curve analysis determined by metabolic growth index (430/560 absorbance rate) comparing cell growth in a Hayflick rich medium (HF) (black) with cell growth in vB13 (red), vB13 without phosphatidylcholine (PC) (purple), vB13 without sphingomyelin (SPM) (blue) and vB13 without both phospholipids (green). **b** Protein biomass measurement at 96 h, corresponding to the end of the growth curve shown in panel A. Data represent the mean of two replicates and error bars the standard deviation.

To show the importance of sphingomyelin and phosphatidylcholine in this medium, growth of *M. pneumoniae* was reported in absence of either one of or both the two phospholipids, as shown in Fig. 6. The lack of growth in absence of both phospholipids and the very poor growth observed when one or the other is missing confirms the essentiality of the concomitant presence of both phosphatidylcholine and sphingomyelin in a serum-free medium for *M. pneumoniae*.

### Effect of hypoxia on *M. pneumoniae* metabolism

**Fig. 7:**
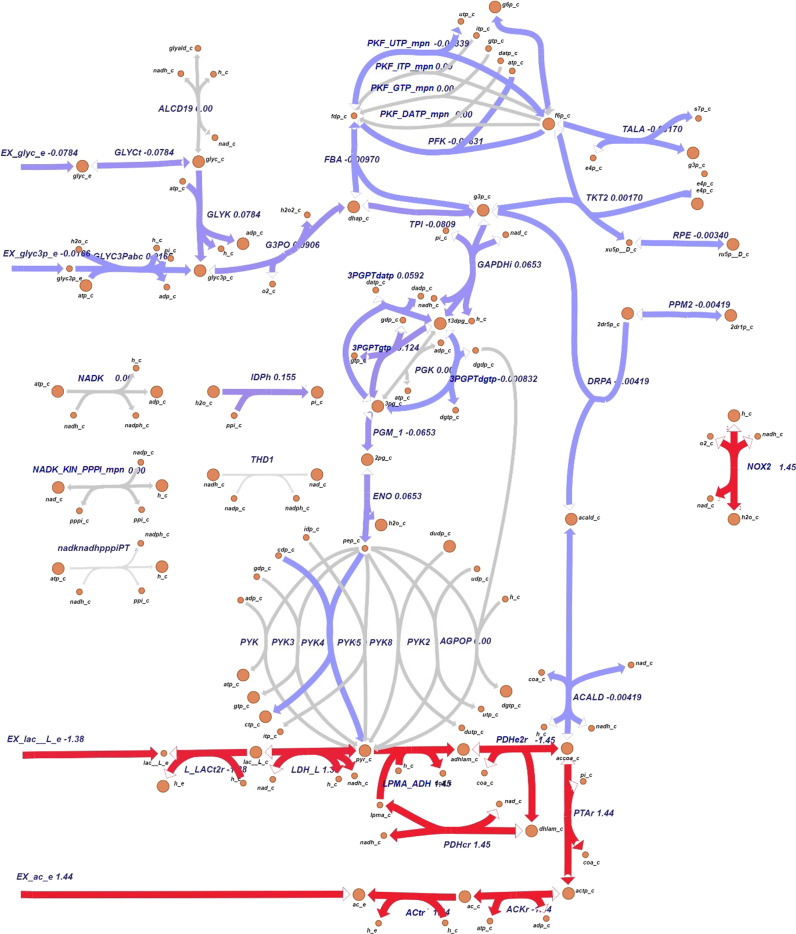
Visualization of flux differences in *M. pneumoniae* glycolysis pathway. Visualization of flux differences in glycolysis reaction when *M*. *pneumoniae* is growing under hypoxia (oxygen uptake 6 mmol.g_DW_^−1^.h^−1^) respect to baseline oxygen availability (7.54 mmol.g_DW_^−1^.h^−1^). Purple is indicative of a small difference (absolute value of flux change <±1) and red of a more considerable one (absolute value of flux change ≥±1). Gray arrows represent reactions that are unused or whose flux does not change when oxygen availability is reduced to 6 mmol.g_DW_^−1^.h^−1^.

**Fig. 8:**
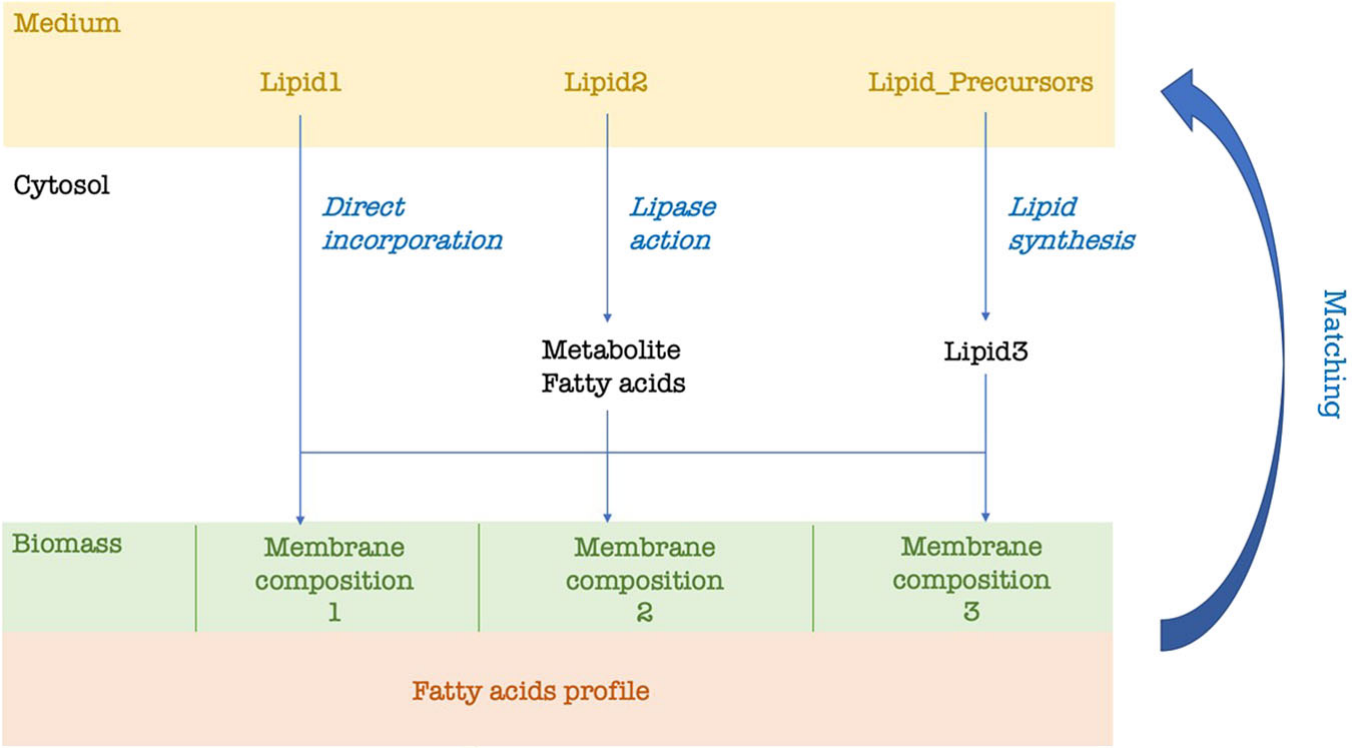
In silico strategy for predicting lipid composition of growth medium. All the possible lipids and precursors are provided in the medium for uptake. Each possible membrane composition that is found in the literature can be incorporated in the model as part of the biomass synthesis reaction. In our case, we considered three membrane compositions whose lipid proportions change according to the cholesterol percentage. Fatty acid profile can be found in literature (as in our model) or be experimentally determined. Lipid1 is known to be directly incorporated into the membrane, Lipid2 is metabolized in the cytosol and, whether a lipase acts, degraded into component head group and fatty acids. Lipid3 is synthetized in the cytosol from lipid precursors. Each lipid is integrated into the membrane according to the compositions previously reported in the literature. According to the reported membrane composition and fatty acid profile, a consensus minimal lipid composition of the medium is predicted.

We used Escher to visualize change of fluxes when simulating different environmental conditions: Fig. 7 shows flux changes when oxygen availability is reduced, as no growth occurs in complete absence of oxygen^45^. With no oxygen limitation and glucose uptake rate of 5.11 mmol.g_DW_^−1^.h^−1^, the model predicts that *M. pneumoniae* takes up 7.54 mmol.g_DW_^−1^.h^−1^ of oxygen and no growth for oxygen uptake rates lower than 4.81 mmol.g_DW_^−1^.h^−1^. The growth rate when oxygen is limited to 6 mmol.g_DW_^−1^.h^−1^ is limited by about 2.3-fold. FVA results for all the reaction fluxes in baseline and hypoxia conditions are reported in Dataset 1, where the reactions included in Fig. 8 are highlighted. Here the fluxes are represented in terms of differences between hypoxia and baseline conditions, given that the solutions for the flux distributions are unique. Despite the overall fluxes’ distribution being slightly reduced under oxygen limitation, the number of zero-flux reactions in this condition is 84. Excluding these, 299 reactions have the same minimum and maximum allowed fluxes. The uniqueness of these solutions allows direct comparison of both conditions.

Not considering the zero-flux reactions, only 18 show the same range of fluxes comparing the two situations (baseline and reduced oxygen) in iEG158_mpn, assuming proton leakage and maintenance energy (ID: “*ATPM*”) do not change. All the lipid synthesis reactions show a consistent flux reduction, as well as the reactions related to the carbon degradation pathways and the NAD kinase. Imports of some amino acids and nucleobases (i.e., thymine, ID: “*THMt6*”) show a flux decrease when oxygen is reduced. Contrarily to the general tendency, deoxyuridine kinase and phosphorylase show a higher flux respect to the baseline situation. CO_2_, H_2_O_2_, acetic acid, and lactic acid production increase under hypoxia.

The whole-metabolism visualization of reaction flows under baseline conditions and when oxygen availability is reduced are reported in Dataset 2.

## Discussion

Owing to its fastidious requirements, *Mycoplasma pneumoniae* has been thus far reported to grow on media containing serum^46,47^ and only poorly in defined medium^18^, furthermore requiring daily changes. The absence of serum elements in the culture medium facilitates the large-scale production of *M. pneumoniae* for medical and pharmaceutical purposes, thanks to the reduced costs, greater reproducibility, definite composition and lack of unexpected serum-plate-agglutination (SPA) antigens^15,48^ and other unpredictable contaminants.

Hereby, by using in silico modeling approaches based on literature data, we propose that sphingomyelin and phosphatidylcholine are essential growth factors allowing *M. pneumoniae* to grow on serum-free media. This prediction was validated by (i) adding these two phospholipids, together with cholesterol and fatty acids, to the previously developed MC medium^47^. This led to the development of a medium called MCMyco, supporting robust *M. pneumoniae* growth; and (ii) removing sphingomyelin and phosphatidylcholine from the defined serum-free medium vB13 (Burgos R., Garcia-Ramallo E., Shaw D., Lluch-Senar M. and Serrano L.), showing the absence of either both phospholipids or one of the two causes lack of growth. Therefore, our study shows, theoretically and experimentally, that supplementation of sphingomyelin and phosphatidylcholine to serum-free media, together with cholesterol, enables growth of *M. pneumoniae*. Moreover, in agreement with the model, we have shown in vB13 that the effect of the two phospholipids is synergistic and that the different incorporation of these lipids depends on their availability in the medium. Indeed, the lipid composition of the membrane might be strongly variable according to the environmental conditions, which affect its permeability^49^ and curvature^50^; for instance, a sphingomyelin-cholesterol packing results in a very tight membrane^51^. Therefore, the key medium lipids herein found confirm that one of the main limitations of *M. pneumoniae* growth resides in its membrane composition, viscosity and permeability. This is, in turn, determined by the proportion of acyl chains in the membrane lipids. While the requirement of cholesterol for growth have been well known for decades^28,52^, phosphatidylcholine supplementation in a medium for *M. pneumoniae* was first reported in 2009^18^. Sphingomyelin, in contrast and to the best our knowledge has not been specifically reported as to be an essential component in media deployed to grow *M. pneumoniae*, although its positive effect on growth was previously suggested in other Mollicutes^53^ and observed in Spiroplasma^54^.

Having established that lipids supplementation is a key aspect for *M. pneumoniae* growth, the way they are supplemented might be critical: *M. pneumoniae* cultures where lipids are supplemented as cholesterol-phosphatidylcholine vesicles show a similar growth rate of the ones in serum-rich media^55^.

Medium composition is not the only growth-limiting factor of *M. pneumoniae* growth in vitro: batch cultures may result in limitation in oxygen supply as a result of gradients that arise due to biofilm formation. Our analysis shows oxygen level is critical, affecting the whole metabolism, with only a few reaction fluxes that remain intact under hypoxic conditions in respect to baseline conditions. Low oxygen not only pushes the lactate dehydrogenase towards the production of lactate, but also critically affects the whole glycolytic pathway, with a reduction of sugar uptake and ATP production. More NADH is consumed and less is regenerated by the GADPH enzyme, which represents one of the main bottlenecks of the metabolic network. The production and accumulation of compounds such as lactate and acetate augments, enhancing toxicity and acidifying the medium when exported: it is observed that the doubling time of *M. pneumoniae* in batch culture increases over time due to the decrease in media pH^11^ and the accumulation of toxic waste compounds, namely acetic and lactic acid. Cells growing in an acidified medium have a different membrane composition^31^. It is, however, still unclear whether the adaptability of *M. pneumoniae* membrane resides in its genome-coding function, if it is a chemical-physical response of the lipid bilayer to pH changes or if it is a combination of these two aspects.

The cytosol of Mycoplasmas does not maintain a constant pH, with the risk of being unable to maintain the proton gradient required for trans-membrane transport^56^ and to alter the internal biochemical state. Our model iEG158_mpn improves iJW145 by not only integrating the membrane, the specific lipids with their pathways and carried acyl chains, but also rearranging the ATP distribution in the maintenance energy, taking into account the effort made by *M. pneumoniae* while de-acidifying the cytosol: a cytosolic pH decrease is equivalent to an increase in the H_3_O^+^ intracellular concentration, which might alter reactions involving H^+^ as reactant^57^. This might be a cause of high energy maintenance costs and reduced growth rate. For this reason, a study on the effects of the intracellular pH change on the metabolism of *M. pneumoniae* would be of main interest.

A study of *M. pneumoniae* under different environmental conditions would not only provide insights into the way it reacts to pH changes, but also disclose the strategies bacteria with a reduced genome use to exploit the host’s resources to accomplish essential tasks, whose capabilities it has lost during the course of evolution: our analysis highlights a lack of metabolic versatility, reflected by an in silico deficiency of flux variability, possibly due to an extremely reduced metabolic network size.

Altogether, our model provides the framework for an integrated approach, incorporating available literature data to systematically discover dependencies that are not of immediate recognition, such as relevant synergies and interconversions, metabolic bottlenecks, cofactor regeneration and redox balancing. Pinpointing knowledge gaps and essential pathways, it demonstrates the usefulness of modeling resources for exploring the metabolic capacity of mycoplasmas, and the use of these insights to rationally influence, modulate and design conditions for optimized performance.

## Methods

### Reconstruction of the genome-scale, constraint-based metabolic model

The metabolic model iJW145^11^ was extensively updated, manually curated and expanded to generate the genome-scale metabolic model iEG158_mpn. Reactions and metabolites have been annotated adding BiGG Models identifiers^58^ when possible. Reaction reversibility has been assessed through eQuilibrator^59^, a thermodynamic calculator of the Gibbs energy released by a specific reaction at a set pH and ionic strength (in our case, respectively, 7.0 and 0.1 M). Reactions where changes in Gibbs energy comprised between −30 and 30 kJ/mol were considered reversible, otherwise irreversible. All model reactions have been manually verified using information from the MyMpn database^60^. Information gathered from literature was used to modify the biomass synthesis reaction adding detailed information on lipid composition. The biomass equation of iEG158_mpn has been standardized to obtain a molecular weight of 1 g.mmol^−1^. Reactions for fatty acid integration into membrane lipid chains have been added. The genome-scale model iEG158_mpn is provided in Dataset 3 in SBML format and its syntax and consistency has been checked with SBML Validator^61^ and MEMOTE, which reported stoichiometry consistency 99.1%, charge balance 87.8% and metabolite connectivity 97.7%^62^. A list of all reactions and metabolites IDs is given in Dataset 4. The model has been deposited in BioModels^63–65^ and assigned identifier MODEL1912060001. A python script to simulate growth with this model, imposing constraints and supplementing in silico the medium, is provided as Dataset 5.

### Model simulations and medium design

Pathways and whole-metabolism maps for fluxes visualization were constructed with the tool Escher^66^, which graphically depicts a solution for maximum attainable growth rate, as computed by Flux Balance Analysis (FBA), with biomass yield, as a proxy for growth, as the objective function^67^. FBA solves a linear programming problem, which is characterized by having more variables than equations, meaning more than one optimal solution can exist for the same objective function maximization. FBA computes a possible flux distribution leading to the optimal objective solution. The same optimal growth rate might be reached by several different reaction flux combinations. Flux Variability Analysis (FVA) explores the range of each metabolic flux at the optimal solution^68^. Both FBA and FVA were run with biomass synthesis reaction as objective function to maximize, respectively, through the commands *optimize()* and *flux_variability_analysis()* of the Python tool *cobrapy*^69^, version 0.5.11 (Python version 3.4.4). Exchange reactions in a genome-scale metabolic model allow metabolites to enter and egress the in silico network. Simulations with iEG158_mpn were performed by conserving the same flux constraints as have been experimentally determined: these assumed unlimited availability of the compounds which are not directly metabolized for energy or carbon and/or cannot be measured experimentally (vitamins, cofactors). Additional fatty acids that could be metabolized or integrated as membrane lipid chains (palmitic acid, palmitelaidic acid, stearic acid, oleic acid, linoleic, and linolelaidic acid) have also been assumed to have unlimited availability as no related uptake rates were experimentally measured. Specifically, exchange reactions corresponding to metabolites whose uptake or secretion rate was not measured were set with bounds of ±1000 when production and secretion was possible, with a lower bound of −1000 and an upper bound of 0 when only uptake from medium was possible and with a lower bound of 0 and an upper bound of 1000 when describing secretion.

Growth medium optimization consists of changing the bounds of exchange reactions to enable or limit uptake or secretion in order to find the combination of supplemented compounds that maximizes the flux through the biomass reaction if this corresponds to maximizing growth rate. We tested the possible uptake of additional native compounds not previously considered in *M. pneumoniae* growth media, but for which transporters are present in *M. pneumoniae* genome, by adding the corresponding exchange reactions. To equilibrate the energetics in the model, we assigned a cost of 1 proton for the uptake of uncharged compounds, 1 ATP for the uptake of compounds with a charge of 1 or 2 and 2 ATPs for the one of compounds with charge greater than 2. To design a minimal serum-free chemically defined medium that optimizes *M. pneumoniae* growth, we iterated through all the exchange reactions present to find the combination of bounds that lead to the highest biomass production: we have initially set all the exchange reactions’ lower bounds to −1000, allowing the metabolites of interest into the system. All the lower bounds of the exchange reactions were set to 0 one at a time, to be then repristinated to −1000 for the next iteration (next exchange reaction to close). When biomass yield was not changing in consequence, we assumed the component was not required for maximizing growth rate. Each time a component was removed from the list, a new set of iterations was started on all the other components. The minimal medium was obtained once all the remaining components, when removed (i.e., when the correspondent exchange reaction’s lower bound was set to 0), were changing the biomass yield obtained by FBA. The relation between the growth rate and the doubling time has been computed using the exponential function e^µT^ = 2, where *µ* is the growth rate in divisions per unit of time and *T* is the doubling time.

To simulate hypoxia, we first ascertained, through Flux Balance Analysis, the minimal oxygen uptake rate required for optimal growth by iEG158_mpn. By allowing in silico an infinite supplementation of oxygen (“EX_O2_e” reaction lower bound = −1000), we ran FBA towards flux maximization of the biomass reaction. Constraining the growth rate to the maximal one, following FBA with objective function “EX_O2_e” (minimization of oxygen uptake), we computed the minimal oxygen required. Once this value is established as the minimal oxygen uptake required for optimal growth, we reduced the oxygen supplementation (i.e., subsequent increments of “EX_O2_e” reaction lower bound from maximum oxygen uptake rate in baseline conditions towards 0) until reaching a biomass flux of 0, meaning no growth in silico. Following this approach, we extracted the minimum required oxygen to be supplemented in silico to allow growth. We then performed simulations of iEG158_mpn with an intermediate oxygen supplementation resulting from the rounded calculation of the mean of the minimal oxygen uptake required for optimal growth and the minimal oxygen required for allowing growth.

### In silico prediction of growth medium lipids based on membrane composition

Lipid composition of the medium was predicted according to the scheme in Fig. 8. Each candidate medium lipid was integrated into the cytosol and subsequently into membrane compartment of biomass in three possible alternative ways: through direct integration, after being metabolized or after synthesis from precursors in the medium. Each lipid is considered to carry fatty acids that match experimentally determined fatty acids profile. Several proportions distributions of the membrane composition were considered, according to the ones detected in literature (as in our model: see Supplementary Table 1). Determination of required composition of lipids in the growth medium (see previous section) is then performed balancing both obtained fatty acid profile and variable membrane compositions, in order to predict a consensus medium composition able to allow growth under all the membrane configurations detected during laboratory cultivation.

### Sequence alignment

By querying manually curated sequences in the UNIPROT database^70^, we verified the presence of lactate and acetic acid transporters and general monocarboxylic acid transporters in the *M. pneumoniae* genome (Dataset 6). PSI-BLAST algorithm^71^ was run with default parameters until convergence was reached to build a sequence profile of the transporters (Position Specific Scoring Matrix provided in Dataset 6), then used to query within *M. pneumoniae* genome for presence of these transporters. In addition, we searched for monocarboxylic transporters in *M. pneumoniae* by running a BLASTp search with default parameters on all its transmembrane proteins, whose list was available on the *MyMpn* portal (August 2018)^60^, filtering for proteins containing transmembrane helices.

### Addition of predicted phospholipids into the semi-defined serum-free medium MCMyco

*M. pneumoniae* strain M129 was grown in 75cm^2^ Tissue Culture Treated Flasks (Falcon) in MCMyco medium, a serum-free medium based on the MC medium previously used for growing *Mesoplasma florum*^44^. Palmitic acid, oleic acid and cholesterol were added at a final concentration of 16.5 µg ml^−1^, 20 µg ml^−1^, and 20 µg ml^−1^, respectively. Supplementary Table 4 reports instructions for MCMyco. Cells were grown and passaged three times in absence or presence of sphingomyelin and phosphatidylcholine at 20 µg ml^−1^ before performing any analysis. Each passage was performed by diluting the cells 50 times in fresh serum-free medium after two weeks of culture. Medium was replaced by fresh serum-free medium every 7 days.

Passage 3 cells DNA were measured by qPCR using oligonucleotides MPN628/5 (GCCATTTTGGATGGTTATGG) and MPN628/3 (GGTGACCCACTTCCGAGTTA) and the Luna® Universal qPCR Master Mix (New England Biolabs). The DNA quantification was performed using the LightCycler® 480 software (Roche). Cells were diluted in 15 ml to an equivalent of 0.6 pg ml^−1^ of DNA and distributed in aliquots of 0.2 ml in 96-well Tissue Culture Treated flat plates (Nunc). Every 12 h, the medium from 6 wells were recovered in 1.5 ml tubes and centrifuged 15 min at 12,000×*g*. 0.2 ml of nuclease-free water were added to the empty wells to lyse adherent cells by osmotic shock and then recovered and added to the previous pellet to lyse non-adherent cells. Finally, they were incubated 10 min at 98 °C and stored at −20 °C. After 120 h, the DNA of each sample was measured by qPCR as described above and the growth curve was then plotted and analyzed using the R software (version 3.4.4).

### Contribution of predicted phospholipids into the defined serum-free medium vB13

*M. pneumoniae* strain M129 was grown in the defined serum component-free medium vB13, developed by Burgos R., Garcia-Ramallo E., Shaw D., Lluch-Senar M. and Serrano L., based on the medium defined by Yus et al.^18^ by experimental screening of different components. vB13 composition is provided in Supplementary Table 5. Growth performance of *M. pneumoniae* in vB13 was compared to rich medium (Hayflick) by growth curve analysis as follows. *M. pneumoniae* strain M129 was inoculated with frozen stocks (1:200 dilution) in tissue culture flasks of 25 cm^2^ containing 5 ml of medium. Cultures were incubated at 37 °C under 5% CO_2_, and protein and DNA biomass were measured at different time points (0 h, 24 h, 48 h, 72, 96 h) as follows. For each time point, cells were scraped off from the flasks in the culture medium and 1 ml of cell suspension harvested by centrifugation (13,100×*g*, 10 min). This was performed per duplicate to obtain samples for both, protein and DNA measurements. For protein biomass quantification, the cell pellet was washed twice with PBSx1 and lysed with 100 μl of lysis buffer (10 mM Tris pH8, 6 mM MgCl2, 1 mM EDTA, 100 mM NaCl, 0.1%Triton-X100 plus cocktail of protease inhibitors) prior to duplicate protein measurements using the Pierce ^TM^ BCA Protein Assay Kit. For DNA biomass quantification, the cell pellet was directly analysed, and the DNA extracted using the MasterPure DNA purification Kit (Epicentre) following the recommendations of the Kit manufacturer. Finally, extracted DNA for each time point was measured using a fluorometric method (Qubit dsDNA HS assay Kit, Invitrogen).

To assess the contribution of phospholipids in the animal component-free medium, *M. pneumoniae* was grown in a 96-well plate format. Growth curve analysis comparing the performance of rich medium (Hayflick), vB13 and vB13 derivative versions containing only phosphatidylcholine (PC), only sphingomyelin (SPM) or vB13 without PC and SPM were recorded in a Tecan Spark plate reader by determining the growth index value, which is the ratio of absorbance at 430 nm and 560 nm of the culture medium^18^. Each media was tested in duplicate wells containing 200 μl of medium inoculated with frozen stocks at 1:100 dilutions. Total cell biomass obtained at the end of the growth curve (96 h) was also determined by protein quantification as follows. Briefly, wells of the 96-well plate were washed twice with PBS ×1 and attached cells lysed with 100 μl of lysis buffer (10 mM Tris pH8, 6 mM MgCl2, 1 mM EDTA, 100 mM NaCl, 0.1%Triton-X100 plus cocktail of protease inhibitors). Protein concentrations were then determined using the Pierce BCA protein assay kit by measuring absorbance at 562 nm in a Tecan plate reader^18^.

### Reporting summary

Further information on research design is available in the Nature Research Reporting Summary linked to this article.

## Supplementary information

Supplementary Information

Reporting Summary

Dataset 1

Dataset 2

Dataset 3

Dataset 4

Dataset 5

Dataset 6

## Data Availability

The authors declare that all the data supporting the modeling are available within the paper and its Supplementary Information. The model has been deposited in BioModels and assigned identifier MODEL1912060001.
