# Supplementary material for: Model-driven design allows growth of Mycoplasma pneumoniae on serum-free media
Source: NPJ Syst Biol Appl. 2020 Oct 23;6:33. doi: 10.1038/s41540-020-00153-7 (PMC7584665; doi:10.1038/s41540-020-00153-7)
Supplement: Supplementary file 1 — Supplementary Information [file 41540_2020_153_MOESM1_ESM.pdf]

Model-driven design allows growth of *Mycoplasma pneumoniae* on serum-free media

Erika Gaspari[*1§], Antoni Malachowski[1§], Luis Garcia-Morales[2†], Raul Burgos[3], Luis Serrano[3,4,5], Vitor A. P. Martins dos Santos[1,6], and Maria Suarez-Diez[*1]*

1. Laboratory of Systems and Synthetic Biology, Wageningen University & Research, Wageningen, the Netherlands
2. INRA, UMR 1332 de Biologie du Fruit et Pathologie , F-33140 Villenave d'Ornon , France
3. Centre for Genomic Regulation (CRG), The Barcelona Institute for Science and Technology, Doctor Aiguader 88, Barcelona, 08003, Spain
4. Universitat Pompeu Fabra (UPF), Barcelona, Spain
5. Institució Catalana de Recerca i Estudis Avançats (ICREA), Pg. Lluis Companys 23, Barcelona, 08010, Spain
6. LifeGlimmer GmbH, MMarkelstrasse 38, Berlin, Germany

§ Equally contributing authors

† Present address: Laboratory of Systems and Synthetic Biology, Wageningen University & Research, Wageningen, the Netherlands

***Corresponding authors**

Erika Gaspari, MSc

erika.gaspariwur@gmail.com

Maria Suarez-Diez, PhD

maria.suarezdiez@wur.nl

Supplementary Material

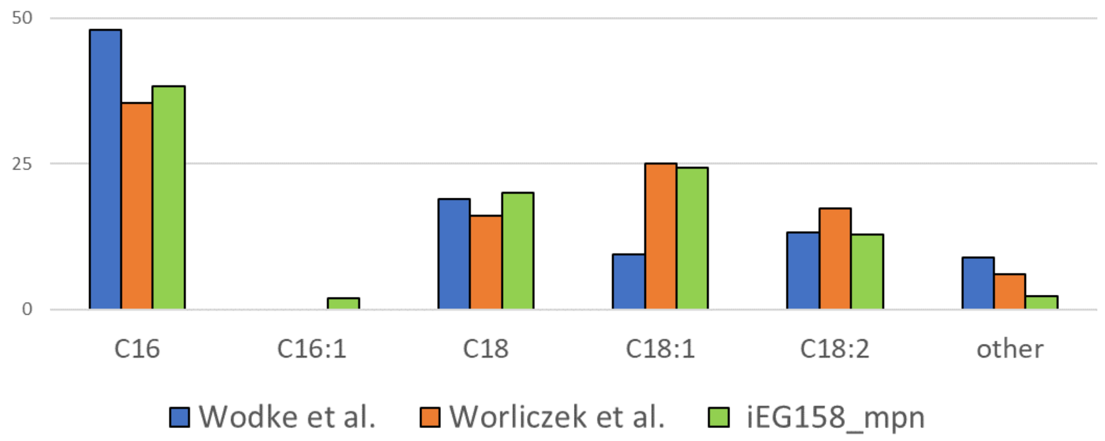

Supplementary Figure S1. *M. pneumoniae* fatty acids composition from different studies.

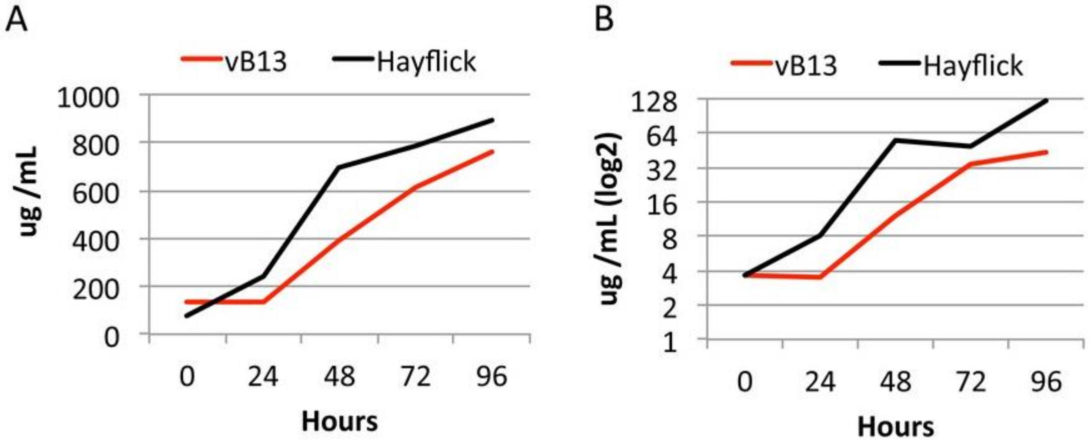

Supplementary Figure S2. Growth curve of *M. pneumoniae* in vB13 medium (defined, animal component-free) compared to Hayflick medium (serum-rich). Cell growth in 25cm 2 tissue culture flasks containing 5ml of medium was assessed by (A) protein and (B) DNA biomass quantification along 96h of culture.

Supplementary Table S1. Different proportions distributions of the lipid membrane composition of *M. pneumoniae* reconstructed from literature.

Lipid	Min Fraction	Max Fraction	Acyl chain	Abs Fraction	Min Rel Fraction	Max Rel Fraction
phosphatidylcholine	0,06	0,1	C16	0,45	0,027	0,045
			C16:1	0,0333	0,001998	0,00333
			C18	0,45	0,027	0,045
			C18:1	0,0333	0,001998	0,00333
			C18:2	0,0334	0,002004	0,00334
cardiolipin	0,15	0,25	C16			
			C16:1			
			C18			
			C18:1	0,25	0,0375	0,0625
			C18:2	0,75	0,1125	0,1875
phosphatidic acid	0,12	0,2	C16	0,75	0,09	0,15
			C16:1			
			C18			
			C18:1	0,25	0,03	0,05
			C18:2			
phosphatidyl glycerol	0,09	0,15	C16	0,75	0,0675	0,1125
			C16:1			
			C18			
			C18:1	0,25	0,0225	0,0375
			C18:2			
sphingomyelin	0,09	0,15	C16	0,15	0,0135	0,0225
			C16:1	0,05	0,0045	0,0075
			C18	0,7	0,063	0,105
			C18:1	0,05	0,0045	0,0075
			C18:2	0,05	0,0045	0,0075
glycolipids	0,06	0,1	C16	0,75	0,045	0,075
			C16:1			
			C18			
			C18:1	0,25	0,015	0,025
			C18:2			
diacylglycerol	0,03	0,05	C16	0,75	0,0225	0,0375
			C16:1			
			C18			
			C18:1	0,25	0,0075	0,0125
			C18:2			
cholesterol	0,35	0,5				

Supplementary Table S2. Results of Flux Variability Analysis on iEG158_mpn.

Supplementary Table S3. All reactions and metabolites in model iEG158_mpn.

Supplementary Table S4. MCMyco serum-free medium detailed composition.

**MCMyco component**	**Volume**
MC base	18 mL
10 g Mycoplasma broth base	
2.5 g glucose	
Sqp 366 mL ph 7.8	
Filter 0.45 µm and 0.22 µm	
+ 83.3 mL yeast extract (Gibco)	
+ 1.25 mL penicillin G 200000 u/mL	
+ 1 mL phenol Red 1%	
Fatty Acids Mix	0.13 mL
0.3 mL palmitic acid (25 mg/mL in etOH)	
0.3 mL oleic acid (30 mg/mL in etOH)	
2.4 mL etOH	
Delipidated Bovin Serum Albumin 4% (Sigma)	1.67 mL
Lipid Mix	0.2 mL
Ethanol 300 µL	
Cholesterol (10 mg/mL) 200 µL	
Sphingomyelin (5 mg/mL) 400 µL	
Phosphatidylcholine (20 mg/mL) 100 µL	

Supplementary Table S5. vB13 serum-free defined medium composition.

Component	vB13
CMRL-1066	0.5X
Glucose	0.75%
HEPES	50mM
Ampicillin	100µg/ml
Phenol red (pH 7)	0.0035%
Glycerol	0.025%
Glutamine	2mM
Spermine	10µg/ml
Phosphathidylcholine	40µg/ml
RNA	1mg/ml
Yeastolate	10mg/ml
cholesterol	30µg/ml
thioctic acid	0.2µg/ml
pyridoxamine	0.5µg/ml
nicotinic acid	0.5µg/ml
riboflavin	0.5µg/ml
choline	0.5µg/ml
sphingomyelin	40µg/ml
2-Hydroxypropyl-β-cyclodextrin	5mg/ml
NaOH (pH7.7)	

Supplementary Methods S1. Pathway visualization maps of *M. pneumoniae* in standard oxygen condition (“baseline”), low oxygen condition (“limited oxygen”) and difference among the two (“differences”). Maps have been constructed with the tool Escher, applied on model iEG158_mpn that was simulated in both standard oxygen condition and reduced oxygen conditions.

Supplementary Methods S2. Genome-scale model iEG158_mpn of *M. pneumoniae*´s metabolism.

Supplementary Methods S3. Python script for simulating *in silico* the growth of *M. pneumoniae* on serum-free medium with model iEG158_mpn.

Supplementary Methods S4. Query of sequences in the UNIPROT database and Position Specific Scoring Matrices used to verify the presence of lactate and acetic acid transporters and general monocarboxylic acid transporters in the *M. pneumoniae* genome.
