# Supplementary material for: Model-driven design allows growth of Mycoplasma pneumoniae on serum-free media
Source: NPJ Syst Biol Appl. 2020 Oct 23;6:33. doi: 10.1038/s41540-020-00153-7 (PMC7584665; doi:10.1038/s41540-020-00153-7)
Supplement: Supplementary file 4 — Dataset 2 [file 41540_2020_153_MOESM4_ESM.gz › Supplementary_Methods1 - Escher/differences.html]

Escher Builder
